# Outcomes of Boston Keratoprosthesis Type I Implantation in Poland: A Retrospective Study on 118 Patients

**DOI:** 10.3390/jcm13040975

**Published:** 2024-02-08

**Authors:** Ewa Wróblewska-Czajka, Dariusz Dobrowolski, Adam Wylęgała, Ula V. Jurkunas, Edward Wylęgała

**Affiliations:** 1 Clinical Department of Ophthalmology, Faculty of Medical Sciences in Zabrze, Medical University of Silesia, 40-760 Katowice, Poland; dardobmd@wp.pl (D.D.); wylegala@gmail.com (E.W.); 2 Ophthalmology Department, Railway Hospital in Katowice, 40-760 Katowice, Poland; 3 Department of Ophthalmology, Santa Barbara Hospital, 41-200 Sosnowiec, Poland; 4 Health Promotion and Obesity Management Unit, Department of Pathophysiology, Faculty of Medical Sciences, Medical University of Silesia, 40-760 Katowice, Poland; adam.wylegala@gmail.com; 5 Cornea Center of Excellence, Schepens Eye Research Institute, Massachusetts Eye and Ear Infirmary, Department of Ophthalmology, Harvard Medical School, Boston, MA 02114, USA

**Keywords:** keratoprosthesis, Boston Type I Kpro, corneal melting, glaucoma, retroprosthetic membrane

## Abstract

**Background:** Boston Keratoprosthesis Type I (BI-KPro I) is a synthetic cornea that can be used to restore vision in patients with corneal blindness. This retrospective study evaluated the outcomes of BI-KPro implantation in 118 patients. Material: The mean age of the patients was 56.76 ± 14.24 years. Indications for keratoprosthesis implantation were as follows: graft failure, 47 (39.83%); ocular burn, 38 (32.20%); neurotrophic keratopathy, 11 (9.32%), mucous membrane pemphigoid 9 (7.67%); autoimmune, 6 (5.08%); Stevens–Johnson syndrome, 4 (3.39%); and aniridia (2.54%). **Methods:** The surgeries were performed between March 2019 and June 2022 at a single clinical center in two locations. The postoperative visual acuity, complications, and need for additional surgical procedures were analyzed. **Results:** The Best Corrected Visual Acuity before surgery was 0.01 ± 0.006. After one year (V1), it was 0.30 ± 0.27; at two years (V2), it was 0.27 ± 0.26; and at three years (V3), it was 0.21 ± 0.23. The percentage of patients with visual acuity better than 0.1 on the Snellen chart was 37.29% after 1 year, 49.35% after 2 years, and 46.81% after 3 years of follow up. The most common complications were glaucoma (78 patients; 66.1%), corneal melting (22 patients; 18.6%), and retroprosthetic membranes (20 patients; 17.0%). **Conclusions:** The BI-KPro can significantly improve visual acuity. The worst long-term results were obtained in the group of patients with autoimmune diseases; therefore, careful consideration should be given to implanting BI-KPro in this group. The high incidence of de novo glaucoma or the progression of pre-existing glaucoma suggests the need for careful monitoring.

## 1. Introduction

Corneal blindness, affecting millions worldwide [1], is typically treated with corneal transplantation. However, limitations such as donor tissue availability and graft rejection necessitate alternatives like Keratoprosthetics (KPro). The Boston Type I KPro (BI-KPro), approved by the FDA in 1992, is a widely used artificial corneal device [2]. It is composed of a front plate, back plate, and a titanium-locking c-ring, and has been shown to improve visual acuity and quality of life in patients with various conditions [3]. However, it is associated with complications like retroprosthetic membrane formation, glaucoma, and corneal melting, necessitating careful patient selection and management [4].

Since the first BI-KPro implantation at the Clinical Department of Ophthalmology of Silesian University in Katowice in 2011 [5], its use has expanded due to full reimbursement by the National Health Fund in mid-2019.

This study reports on the outcomes of BI-KPro implantation in 118 patients over a three-year follow-up period.

## 2. Materials and Methods

A retrospective review of the surgical treatment outcomes was conducted in 118 patients, comprising 46 females and 72 males, who underwent BI-KPro implantation.

The participants had an average age of 56.76 ± 14.24 years.

Prior to the procedure, 65 patients underwent at least one corneal transplantation, 22 underwent limbal grafting, and 9 underwent Ahmed valve implantation. K-pro implantation was combined with cataract extraction in 37 patients and intraocular lens (IOL) explantation in 58 patients. Twenty-three patients were aphakic prior to the procedure. In one case, K-pro was implanted in the phakic eye.

Indications for keratoprosthesis implantation are presented in Table 1.

The follow-up period of the patients after the procedure ranged from 12 to 39 months, with a mean of 24.8 ± 9.20.

All surgeries were performed by two surgeons between March 2019 and June 2022. Patients were recruited from a single clinical center in two locations: the Clinical Department of Ophthalmology at Silesian Medical University in the District Railway Hospital in Katowice, and the Ophthalmology Department of St. Barbara’s Hospital in Sosnowiec.

The medical history of all patients who qualified for keratoprosthesis implantation was analyzed and a full ophthalmic examination was performed. Best-corrected visual acuity and light projection were determined. Intraocular pressure (IOP) was measured depending on the clinical condition of the eye using Goldman’s applanation method, the iCare device, or palpation. Slit lamp examination with photographic documentation, Schirmer’s test, A- and B-scan ultrasound, and anterior segment optical coherence tomography (OCT) were performed. The criteria used to determine the candidacy of keratoprosthesis surgery were in accordance with The Boston Keratoprosthesis International Protocol.

Postoperatively, data on the BCVA (Best Corrected Visual Acuity), presence of complications, coexisting diseases, and need for additional surgical procedures were analyzed.

All patients provided informed consent prior to any surgical intervention.

This retrospective observational study, in accordance with Polish law, did not require approval from the local ethics committee.

### 2.1. Keratoprothesis Implantation Technique

Prior to the procedure, an 8.5-mm-diameter corneal button was excised from the donor cornea, and a 3-mm-diameter trephine was used to create a central opening in the button. The optical parameters of the keratoprosthesis were determined based on the patients’ axial length measurements obtained during preoperative evaluation. The prepared donor corneal button, titanium plate, and securing ring were sequentially placed on the optical cylinder of the keratoprosthesis to ensure a tight fit between the components. All procedures were performed under general anesthesia. If necessary, adhesions between the eyelids and globe were released into the recipient’s eye, followed by the excision of the cornea using an 8 mm trephine. If the patient was not aphakic, the crystalline lens or intraocular lens was removed, except in one case in which the keratoprosthesis was implanted while preserving the patient’s own lens. Peripheral iridotomy was performed in the patients with significant anterior synechiae. Anterior vitrectomy was performed in cases of rupture of the posterior capsule. The keratoprosthesis was secured using single 9.0 or 10.0 nylon sutures.

After the procedure, a soft contact lens was inserted and the following eye drop regimen was recommended: antibiotic drops every 1 h, steroid drops every 1 h, moisturizing drops every 1 h, and a mydriatic agent three times a day. Pressure-lowering drops were added if necessary. Steroids and antibiotics were typically used. Topical cyclosporine and/or systemic mycophenolate mofetil is recommended for patients with autoimmune diseases. The dosage of medications was modified based on clinical conditions. For all patients post-procedure, a daily regimen of antibiotic eye drops three times a day, mydriatic eye drops once a day, and artificial tears, at a minimum, every two hours was advised. In cases where contact lenses provided comfort, facilitated the retention of the intact corneal epithelium, and improved the quality of vision, their use was continued indefinitely. The majority of contact lenses used were replaced on a monthly basis.

### 2.2. Statistical Analysis

Statistical analyses were performed using Statistica v13 (Tibco, Palo Alto, CA, USA). Descriptive statistics were used to summarize the demographic characteristics of the study population. Qualitative measurements were compared using the chi-squared test. Quantitative measurements were analyzed using the Mann–Whitney U test, repeated measures ANOVA, and post hoc least significant difference (LSD) test to evaluate differences between groups. Statistical significance was set at *p* < 0.05.

## 3. Results

### 3.1. Visual Acuity

Visual acuity measurements were obtained using a standard Snellen chart.

Visual acuity measurements, recorded as counting fingers, hand motion, and light perception, were converted to Snellen equivalents using the conversion algorithm described by Holladay.

The statistical analysis included BCVA before the procedure and results obtained one year (V1), two years (V2), and three years (V3) after the procedure.

**Figure 1:**
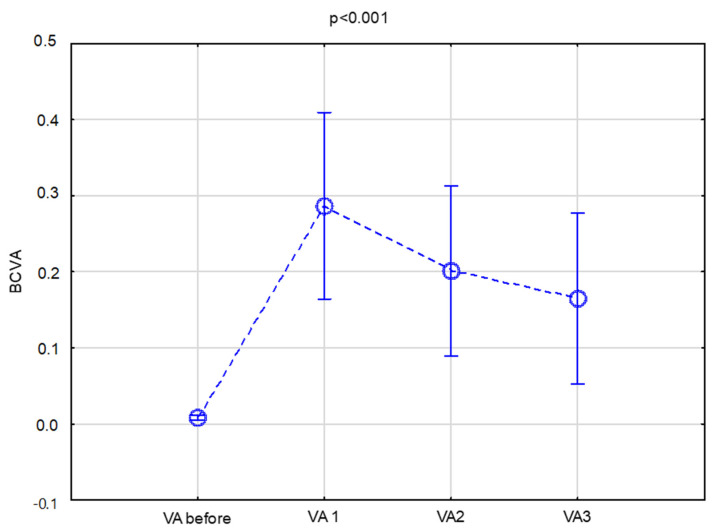
Visual acuity outcomes in the overall study population at baseline and 1, 2, and 3 years after BI-KPro implantation.

The mean BCVA before surgery was 0.01 ± 0.006. After V1, it was 0.30 ± 0.27; at V2, it was 0.27 ± 0.26; and at V3, it was 0.21 ± 0.23 (Figure 1). Post hoc LSD tests were conducted to compare the means of the different groups for BCVA. There were significant differences between the VA before and after surgery for all timepoints (*p* < 0.001). There were also significant differences between V1 and VA2 (*p* = 0.004634) and between VA2 and VA3 (*p* = 0.00937).

**Figure 2:**
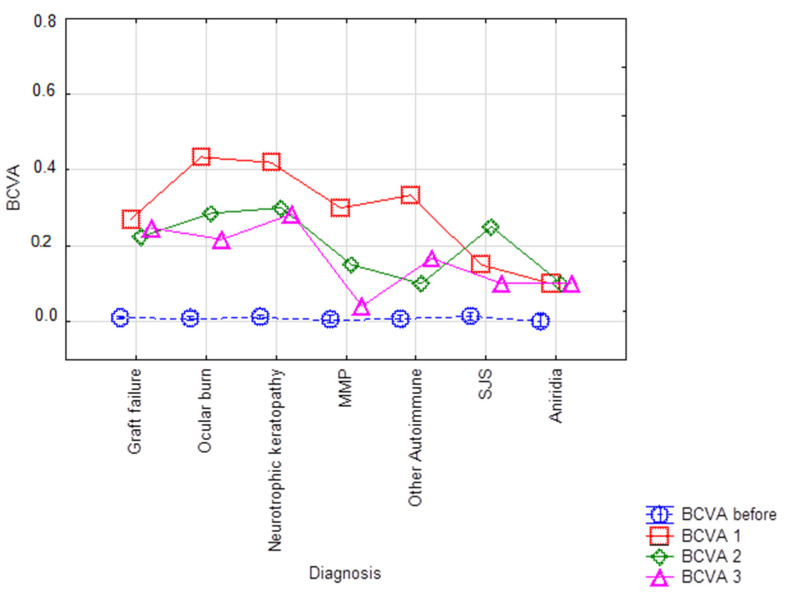
Visual acuity outcomes by indication at baseline and 1, 2, and 3 years after BI-KPro implantation.

Transplant indications led to different visual outcomes among the groups, with graft failure, ocular burns, and neuropathic keratopathy achieving the highest BCVA three years after the procedure, whereas pemphigoid had the lowest (Figure 2).

**Figure 3:**
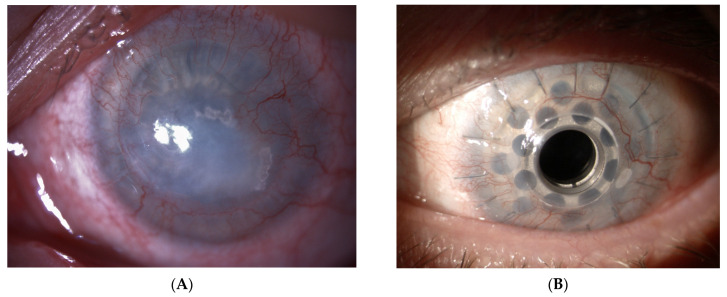
(**A**) Slit-lamp photograph of the right eye of a 37-year-old female patient with a history of chemical burn to the ocular surface, 360-degree allogeneic corneal limbal graft, and two penetrating keratoplasty procedures. The patient’s visual acuity is CF. Visible corneal leucoma, vascularized from the periphery, with the outline of the iris. (**B**) The same eye 18 months after BI-KPro implantation; visual acuity with correction of −1.0 D sph is 0.8. A properly positioned BI-KPro with a partially vascularized corneal component is visible.

The percentage of patients in the entire study group who achieved visual acuity better than 0.1 on the Snellen chart was 37.29% after one year, 49.35% after two years, and 46.81% after three years of follow up (Figure 3A,B).

**Figure 4:**
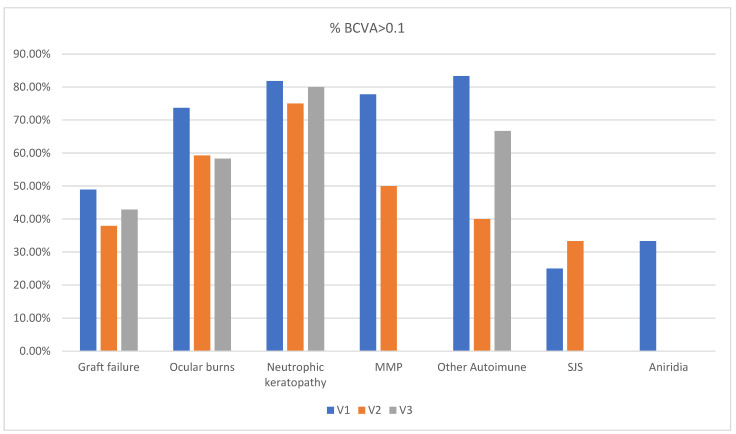
The percentage of patients with visual acuity better than 0.1 after 1 year, 2 years, and 3 years from surgery, according to the indication for transplantation.

The percentage of patients with visual acuity better than 0.1 after 1 year, 2 years, and 3 years from surgery, according to the indication for transplantation, is shown in Figure 4 and Table 2.

### 3.2. Complications

#### 3.2.1. Glaucoma

At baseline, 54 patients had glaucoma, and glaucoma was detected in 78 patients after the procedure (*p* < 0.01). In eight patients diagnosed with glaucoma before keratoprosthesis implantation, there was a need to add at least one additional antiglaucoma medication. Glaucoma surgery was required in seven eyes. Intraocular pressure was determined using palpation.

#### 3.2.2. Corneal Melting

**Figure 5:**
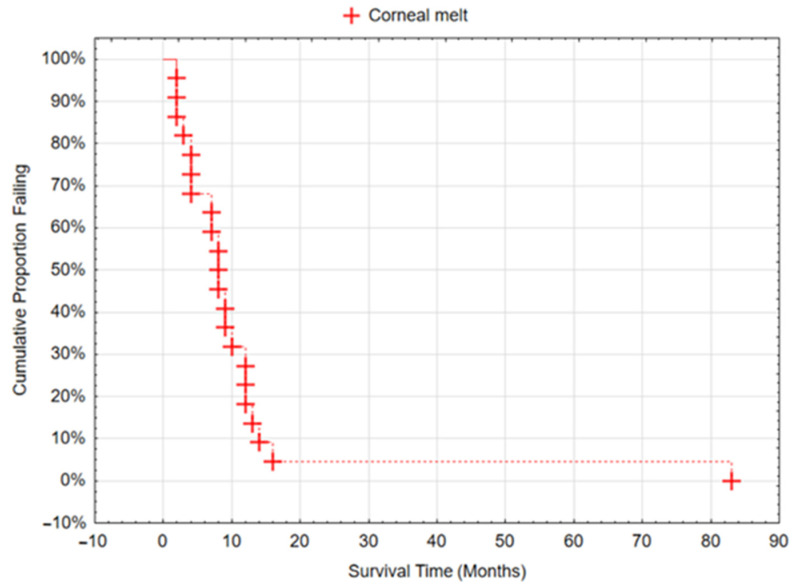
Corneal melting as a function of time after BI-KPro implantation.

In a case series of 118 eyes, the frequency of corneal melting after the implantation of the Boston Keratoprosthesis was 18.6% (22 eyes). The shortest time for this complication to appear was 2 months, while the longest was 21 months. The median time to corneal melting was 11.3 months ± 16.5 (Figure 5).

If a patient presented with mild symptoms, the diagnosis of keratomalacia resulted in the modification of treatment in the form of the application of a soft contact lens in patients who did not use it on a daily basis, reduction in steroid drops, and inclusion of oral doxycycline 2 × 100 mg per day.

Additional surgical procedures were performed in cases where the applied treatment was ineffective or advanced corneal melting was observed at the time of the patient’s presentation to the ophthalmology clinic.

In four cases of superficial changes, an amniotic membrane was grafted onto the surface of the cornea. The symptoms were withdrawn, and full epithelialization was achieved in all patients.

A corneal limbus graft procedure was performed in 14 patients. Depending on the extent of melting, grafting was performed from 45 to 360°.

In nine patients, the limbal graft was supplemented with amniotic membrane patching on the graft and in two patients with a conjunctival flap. Two patients required a second limbal graft during the follow-up period due to recurrent corneal melting. In two cases, three limbal grafts were necessary.

### 3.3. Retroprosthetic Membrane

#### 3.3.1. Retroprosthetic Membrane Was Described in 20 Patients

**Figure 6:**
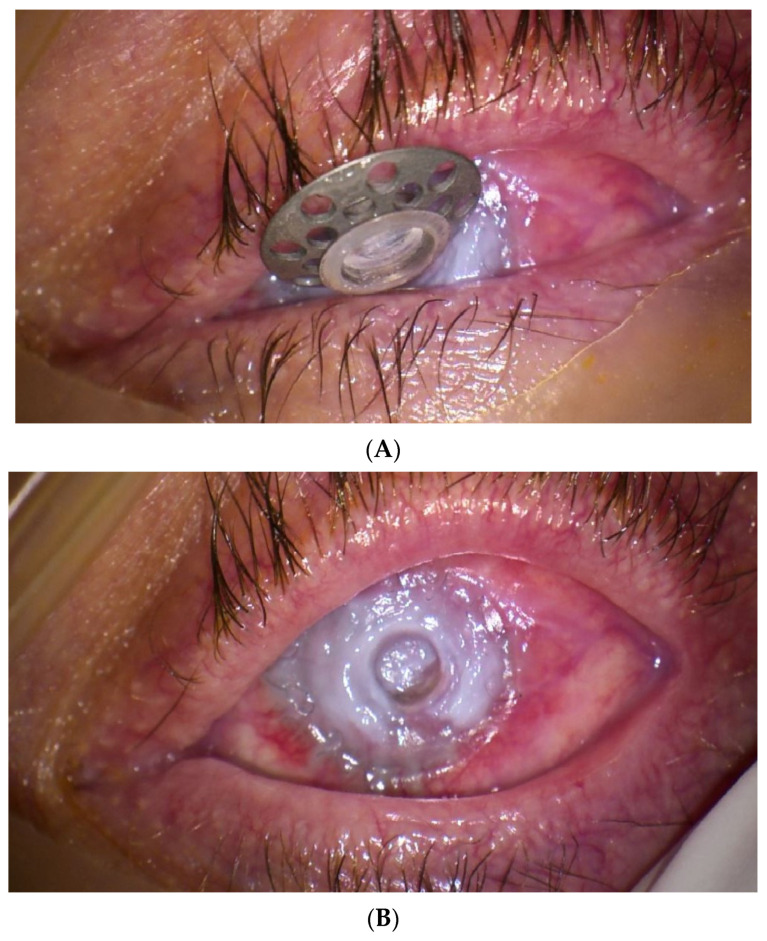
(**A**) Operating microscope photograph of the left eye of a 67-year-old female patient with a history of MMP. She underwent BI-KPro implantation 1 year ago. The corneal prosthesis is spontaneously evacuated. Visual acuity is light perception with full localization. (**B**) A thick, solid, sealed membrane was observed on the entire posterior surface of the corneal prosthesis. No leakage was detected, and the intraocular pressure remained normal.

In 12 cases, it occurred in patients who subsequently developed corneal melting, and in three cases with retinal detachment. In one case, the spontaneous evacuation of the keratoprosthesis occurred with the preservation of the continuity of the eyeball, owing to the formation of a very thick membrane behind the entire posterior surface of the keratoprosthesis (Figure 6A,B).

In 17 cases, Nd:Yag was performed to expose the optical axis; in 3 cases, the membrane was mechanically removed during surgery. In six cases, a repeat procedure with a laser was necessary and in three cases, the membrane was formed three times.

#### 3.3.2. Posterior Capsule Opacification

Posterior capsule opacification (PCO) was described in 11 patients, and Nd:YAG capsulotomy was required in all the cases.

#### 3.3.3. Cystoid Macular Edema

Cystoid macular edema was diagnosed in seven patients, and intravitreal injections were administered in three patients.

#### 3.3.4. Epiretinal Membrane

An epiretinal membrane (ERM) was diagnosed in six patients. Owing to visual acuity deterioration ranging from 2 to 4 rows on the Snellen chart and high surgical risk, the patients were not treated surgically.

#### 3.3.5. Retinal Detachment

Retinal detachment (RD) occurred in three patients. All the patients underwent pars plana vitrectomy.

#### 3.3.6. Vitreous Hemorrhage

Vitreous hemorrhage (VH) occurred in three patients, none of whom required vitrectomy.

#### 3.3.7. Endophthalmitis

Two patients were diagnosed with inflammation in the interior of the eyeball. One patient was treated with general and local antibiotic therapies. In another patient, the inflammatory state was also eliminated after pharmacological treatment; however, after 3 months, there was a relapse, and pars plana vitrectomy was necessary. The number of complications, according to the indications for BI-KPro, is shown in Table 3.

## 4. Discussion

Numerous publications have demonstrated that, in some cases of corneal disease, keratoprosthesis implantation is the only way to achieve useful visual acuity [6]. In this study, a statistically significant improvement in visual acuity was observed compared to the pre-keratoprosthesis implantation state at one, two, and three years postoperatively. However, for the entire study group, the visual acuity after two years was significantly lower than that achieved after the first year. This is because of postoperative complications, particularly corneal melting, retinal detachment, changes in the posterior pole of the eye, and endophthalmitis, most of which occurred before the second year of follow up. The visual acuity achieved was dependent on the indication for the procedure. The greatest improvement after one year was observed in the patients with ocular burns. One of the hypotheses considered was whether this result was due to a tendency toward a less frequent formation of retroprosthetic membranes (RPMs) in such patients, which has also been described by other authors [7]. However, a decline in visual acuity was observed in patients from the presented group in subsequent years, most likely due to glaucoma progression, which occurred before the procedure in all the patients after eye burning.

The worst results caused by macular hypoplasia were obtained in patients with aniridia [8]. In this group, as well as in patients with multiple graft failures, the achieved visual acuity was the most stable and associated with the lowest overall incidence of complications. The worst prognostically in terms of the final visual acuity group were patients with mucous membrane pemphigoid, similar to the reports of other authors [9]. In this group of patients, there was a significant degree of visual impairment affecting both eyes; therefore, sometimes even a slight final improvement is a great help in daily functioning. In the group of patients with Stevens–Johnson syndrome (SJS), better visual acuity achieved after two years than after the first year postoperatively was associated with the performance of Nd:Yag membranotomy due to a membrane that appeared between the first and second years in all patients.

Patients that qualify for keratoprosthesis implantation are often diagnosed with glaucoma due to anatomical changes in the anterior segment after multiple grafts, injuries, or secondary inflammatory conditions such as HSV infection. Additionally, inflammation caused by the procedure itself or a reaction to BI-KPro as a foreign body, changes in the filtration angle, or chronic steroid therapy may cause glaucoma or contribute to its progression.

In the study group, 45.7% of patients were diagnosed with glaucoma before keratoprosthesis implantation. This corresponds to the results of other researchers who showed an earlier diagnosis of glaucoma in 36–76% of patients receiving BI-KPro [10,11,12]. In this group, elevated intraocular pressure requiring the modification of pharmacotherapy or surgical treatment was observed in 27.7% of patients, consistent with previous reports [13,14].

De novo glaucoma after keratoprosthesis implantation was diagnosed in 20.33% of the patients, similar to other studies [13,15,16].

This study also confirmed glaucoma as the main complication of BI-KPro implantation, which requires careful patient examination after the procedure. In particular, an increase in intraocular pressure can be assessed through palpation, which is not precise, even for experienced ophthalmologists.

The method proposed by other authors is scleral pneumometry with an overestimation of the result by approximately 9 mmHg compared with corneal examination or measurement with a Schiotz tonometer in the temporal part of the sclera or near the limbus [17,18]. In addition, the assessment of perimetry in patients after BI-KPro implantation has a reduced diagnostic value owing to the limitation of the visual field to approximately 90–95 degrees [19].

Corneal melting is one of the most serious complications after BI-KPro implantation. It can lead to corneal perforation, hypotony, choroidal and retinal detachments, and endophthalmitis. The current rate of this complication is estimated to be between 11 and 29.5% [20]. In this study, we found that corneal melting occurred in 18.6% of the patients. However, autoimmune diseases are major risk factors for the development of this complication owing to the presence of chronic inflammation. MMP, SJS, and other immunological diseases accounted for 16.10% of all patients in this study. Corneal melting was observed in 44.4% of MMP cases. This correlates with reports, where five of eight patients with MMP required further surgery due to melting [9]. The implantation of BI-KPro in patients after eye burns can be associated with corneal melting in up to 40% of the patients [21]. In this study, the incidence of this complication was 21%. However, as many as 57.8% of patients underwent a corneal limbal transplant before BI-KPro I implantation. This eliminated epithelialization disorders as risk factors for corneal melting. The fact that none of them had the procedure performed before three years had passed since the burn may have also contributed to reducing inflammation.

In patients in this study, after applying pharmacological treatment modifications or amniotic membrane transplantation, improvement was achieved only in patients with shallow and not extensive changes. In the remaining cases, the diseased tissue was replaced with new tissue.

The formation of a membrane behind a keratoprosthesis is a tissue reaction to foreign devices. Boston Type I Keratoprostheses, which use titanium plates instead of PMMA, are less responsible for membrane formation. However, even in these cases, the complication rate is very high, ranging from 25% to 65% [22]. The incidence of complications in this study was 16.9%. Among autoimmune diseases, it occurs most often (33.3% to 100%) in patients with SJS. According to some authors, the highest probability of RKM occurs in patients with a history of corneal infection and aniridia. However, other studies have not described the diagnosis as a risk factor [7,23]. The appearance of the RKM can significantly impair vision by occluding the optical axis. However, it can be restored relatively noninvasively by performing Nd:YAG membranotomies. The development of RKM requires careful monitoring of the eye condition because of the secondary, more serious complications that it can lead to.

RKM can cause corneal melting due to impaired aqueous flow to the cornea and glaucoma through the occlusion of the drainage angle, and can lead to retinal detachment or the extrusion of the keratoprosthesis due to contraction [23]. In this study, 59.09% of the eyes with diagnosed corneal melting and all patients with retinal detachment had RKM. Moreover, the only patient in whom BI-KPro I was lost because of spontaneous evacuation had a very thick membrane that tightly sealed the eyeball under the keratoprosthesis.

Despite the use of chronic antibiotic therapy, endophthalmitis was diagnosed in two of the patients from the presented group, accounting for 1.6% of all patients. This value falls within the range reported by other authors (0–15.5%). In both cases, Staphylococcus aureus was found in the cultures, which also confirmed the identification of Gram-positive bacteria as the main pathogen [24].

Retinal detachment occurred in 2.5% of cases, which is significantly less frequent than that reported in the literature (4.76–15.5%) [25]. A possible reason for this is that in most cases, corneal melting was surgically treated before leakage and hypotony occurred.

The results of this study may serve as a source of information regarding the benefits and risks associated with the implantation of a Boston Type I Keratoprosthesis. However, the interpretation of these results should take into account the limitations of this study. The study involved 118 patients, which, while not small, may not be large enough to detect all differences in outcomes among subgroups of patients with different indications for keratoprosthesis implantation. The follow-up duration was three years. Longer-term outcomes and complications may not be captured within this timeframe. This is particularly in relation to the observed decrease in visual acuity in subsequent years compared to the observations after the first year. The study group remains under observation, and the publication of long-term results is planned for the future.

## 5. Conclusions

This study confirmed that the use of the Boston Keratoprosthesis Type I (BI-KPro I) can improve visual acuity, with the mean Best Corrected Visual Acuity showing substantial improvement from 0.01 ± 0.006 pre-surgery to 0.21 ± 0.23 three years post-procedure. Notably, a significant proportion of patients (46.81%) achieved a visual acuity better than 0.1 on the Snellen chart after three years of follow up.

However, this study also highlighted the importance of appropriate patient selection. The group of patients with autoimmune diseases, especially mucous membrane pemphigoid, showed the worst long-term results and the highest percentage of complications. This suggests that careful consideration should be given before implanting the BI-KPro I in patients with these conditions.

Complications such as glaucoma and corneal melting were observed in a significant number of patients post-procedure, emphasizing the need for careful postoperative management and follow up. The formation of retroprosthetic membranes was another notable complication and was associated with other complications such as corneal melting and retinal detachment.

Further studies are needed to optimize patient selection and postoperative management strategies to maximize the benefits of this procedure.

## Figures and Tables

**Table 1:** Ocular indication for the transplantation.

Category	Count	Percent
Total count	118	100
Graft failure	47	39.83
Ocular burn	38	32.20
Neurotrophic keratopathy	11	9.32
Mucous membrane pemphigoid	9	7.67
Other autoimmune diseases	6	5.08
Stevens–Johnson syndrome	4	3.39
Aniridia	3	2.54

**Table 2:** The percentage of patients with visual acuity better than 0.1 after 1 year, 2 years, and 3 years from surgery, according to the indication for transplantation.

	GF	OB	NK	MMP	OA	SJS	A
V1	48.94%	73.68%	81.82%	77.78%	83.33%	25.00%	33.33%
V2	37.93%	59.26%	75.00%	50.00%	40.00%	33.33%	0.00%
V3	42.86%	58.33%	80.00%	0.00%	66.67%	0.00%	0.00%

V1 = one year, V2 = two years, and V3 = three years after BI-KPro implantation, GF = graft failure, OB = ocular burn, NK = neurotrophic keratopathy, MMP = mucous membrane pemphigoid, OA = other autoimmune disease, SJS = Stevens–Johnson syndrome, A = aniridia.

**Table 3:** Number of complications depending on the indication for BI K-Pro.

Category	Total Count (100%)	CM	RKM	PCO	CMO	ERM	RD	VH	Endophthalmitis	BI-KPro Extrusion
GF	47	6	8	4	3	3		1		
		12.5%	17.0%	8.5%	6.3%	6.3%		2.1%		
OB	38	8	2	5	1		1	2	1	
		0.21	5.2%	13.1%	2.6%		2.6%	5.2%	2.6%	
NK	11	1		2	2	2				
		0.09%		18.1%	18.1%	18.1%				
MMP	9	4	3		1		1			1
		44.4%	33.3%		11.1%		11.1%			11.1%
OA	6	2	3			1	1		1	
		33.3%	33.3%			16.6%	16.6%		16.6%	
SJS	4	1	4							
		0.25%	1							
A	3					1				
						33.3%				
Total count	118	22	20	11	7	7	3	3	2	1
		18.6%	16.9%	9.3%	5.9%	5.9%	2.5%	2.5%	1.6%	0.8%

CM = corneal melting, RKM = retroprosthetic membrane, PCO = posterior capsule opacity, CMO = cystoid macular oedema, ERM = epiretinal membrane, RD = retinal detachment, VH = vitreous hemorrhage, GF = graft failure, OB = ocular burn, NK = neurotrophic keratopathy, MMP = mucous membrane pemphigoid, OA = other autoimmune disease, SJS = Stevens–Johnson syndrome, A = aniridia.

## Data Availability

The data that support the findings of this study are available from the corresponding author, upon reasonable request.
